# Effect of *h*-BN Support on Photoluminescence of ZnO Nanoparticles: Experimental and Theoretical Insight

**DOI:** 10.3390/ma15248759

**Published:** 2022-12-08

**Authors:** Danil V. Barilyuk, Ekaterina V. Sukhanova, Zakhar I. Popov, Artem A. Korol, Anton S. Konopatsky, Dmitry V. Shtansky

**Affiliations:** 1 National University of Science and Technology “MISIS”, Leninsky Prospect 4, Moscow 119049, Russia; 2 Laboratory of Acoustic Microscopy, Emanuel Institute of Biochemical Physics RAS, Kosygina 4, Moscow 119334, Russia; 3 Academic Department of Innovational Materials and Technologies Chemistry, Plekhanov Russian University of Economics, 36 Stremyanny per., Moscow 117997, Russia

**Keywords:** boron nitride, zinc oxide, heterostructures, photoluminescence, DFT

## Abstract

Herein we report a simple and easily scalable method for fabricating ZnO/*h*-BN composites with tunable photoluminescence (PL) characteristics. The *h*-BN support significantly enhances the ultraviolet (UV) emission of ZnO nanoparticles (NPs), which is explained by the ZnO/*h*-BN interaction and the change in the electronic structure of the ZnO surface. When *h*-BN NPs are replaced with *h*-BN microparticles, the PL in the UV region increases, which is accompanied by a decrease in visible light emission. The dependence of the PL properties of ZnO NPs on the thickness of *h*-BN carriers, observed for the first time, is explained by a change in the dielectric constant of the support. A quantum chemical analysis of the influence of the *h*-BN thickness on the electron density redistribution at the *w*ZnO/*h*-BN interface and on the optical properties of the *w*ZnO/*h*-BN composites was carried out. Density functional theory (DFT) calculations show the appearance of hybridization at the *h*-BN/*w*ZnO interface and an increase in the intensity of absorption peaks with an increase in the number of h-BN layers. The obtained results open new possibilities for controlling the properties of ZnO/*h*-BN heterostructures for various optical applications.

## 1. Introduction

Zinc oxide nanoparticles (NPs) have an attractive combination of optoelectronic, antibacterial, and magnetic properties, which make them a promising material in medicine, cosmetics, photocatalysis, solar cells, and photodetectors [1]. The optical properties of ZnO, namely photoluminescence (PL), are of particular interest because they play a crucial role in most of the above areas. ZnO NPs can emit in the ultraviolet (UV) range and in the visible region. Emission in the UV region is called near-band-edge (NBE) emission and it is associated with band-to-band transitions. Emission in the visible region is called deep level emission (DLE) and is related to surface defects [2]. NBE and DLE are two competing processes that can be customized by creating heterojunctions. In particular, the PL of ZnO NPs can be controlled via surface passivation by creating core-shell structures or coatings [3,4,5]. This gives the possibility of creating a versatile material in which the optical properties of ZnO NPs can be finely tuned depending on the application without changing the fabrication process. There is an urgent demand for such a material, because ZnO NPs are increasingly being used in UV light emitting devices (LED) and in cosmetics, but the requirements for the optical properties of ZnO NPs are different. In the first case, one needs ZnO NPs to emit in the UV range (<400 nm), while in the second case UV radiation will be harmful to the skin, so ZnO NPs emitting in the visible range (400–800 nm) are in demand [1,6]. Layered two-dimensional (2D) materials are a promising support for ZnO NPs, because by changing the number of layers, i.e., the thickness, it is possible to change the properties of the 2D material, which may directly affect the optical properties of ZnO NPs [7,8]. In addition, it is easy to deposit ZnO NPs particles on them, which is a prerequisite for large-scale production [9]. Among various 2D materials, *h*-BN has benefits as a support material for ZnO NPs due to its wide bandgap, thermal stability, low dielectric constant and biocompatibility [10]. The optical properties of ZnO/*h*-BN heterostructures are of interest, but the role of *h*-BN in the photoluminescence of ZnO NPs is not clear [11]. In this paper, we report a simple and scalable method for fabricating versatile ZnO/*h*-BN heterostructures with tunable PL characteristics. A noticeable increase in the NBE emission of ZnO NPs is observed with an increase in the *h*-BN thickness. The effect of h-BN thickness on the electron density redistribution at the *w*ZnO/h-BN interface and on the optical properties of *w*ZnO/*h*-BN heterostructures is discussed based on density functional theory (DFT) calculations. Our findings contribute to understanding of the optical properties of ZnO NPs interactions with 2D layered materials, which is important for the development of versatile heterostructures for wide range of applications: from photocatalysis to biomedicine.

## 2. Materials and Methods

### 2.1. Materials

**Figure 1:**
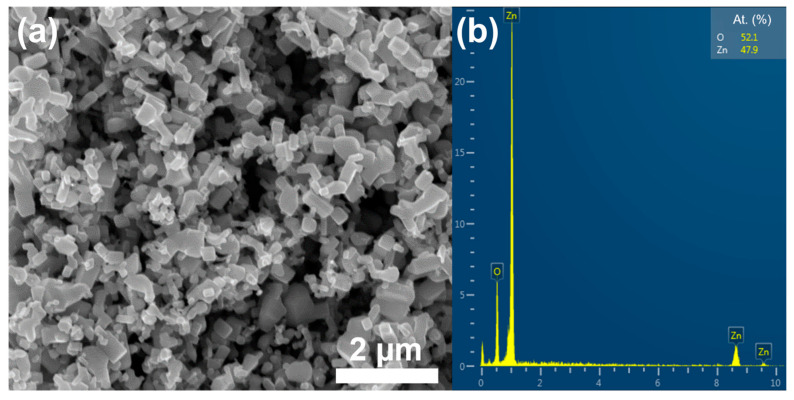
SEM image (**a**) and corresponding EDX spectrum (**b**) of commercial ZnO powder.

Hexagonal *h*-BN platelets (Plazmoterm, Russia) with an average size of ~ 1 µm and ~100 nm (hereinafter referred to as *h*-BNµm and *h*-BNnm, respectively) and ZnO powder (Plazmoterm, Russia) several micrometers in size (Figure 1) were used as starting materials. Zinc acetate dihydrate ((CH_3_COO)_2_Zn × 2H_2_O) was acquired from Lenreaktiv (St Petersburg, Russia). Sodium hydroxide (NaOH) and isopropyl alcohol (IPA) were purchased from PrimeChemicalGroup (Mytishchi, Russia).

### 2.2. Synthesis

ZnO NPs were obtained as described elsewhere [12]. A total of 30 mg NaOH was dissolved in 15 mL IPA and cooled to 0 °C. Then, 100 mg (CH_3_COO)_2_Zn × 2H_2_O was dissolved in 25 mL IPA and then added to 125 mL IPA cooled to 0 °C. Then, the cooled NaOH solution was added slowly with gently stirring. The resulting solution was placed in a thermostat heated to 65 °C and kept for 1 min. After that, 50 mL of the sonicated suspension of h-BN in IPA was added. The resulting suspension was cooled in a thermostat for 1.5 h at a cooling rate of 0.7 °C/min. The heterostructures were subtracted by vacuum filtration using a cellulose acetate filter (d = 0.2 µm) and washed with IPA.

### 2.3. Characterization 

The structure, chemical composition, and distribution of ZnO NPs on the *h*-BN surface were studied by transmission electron microscopy (TEM) and scanning transmission electron microscopy (STEM) using a FEI Technai Osiris 200 kV (FEI company, Hillsboro, OR, USA) equipped with an energy-dispersive X-ray spectrometer (EDXS). The morphology of *h*-BN micro- and nanoparticles was analyzed by scanning electron microscopy (SEM) on a JEOL 7600F (JEOL, Tokyo, Japan) equipped with an X-Max detector (Oxford Instruments, Abingdon, UK). The phase composition of the ZnO/*h*-BN heterostructures was determined by X-ray diffraction (XRD) on a DRON-4 diffractometer (Bourevestnik, Saint-Petersburg, Russia) operating at an accelerating voltage of 40 kV, a current of 19 mA, and Co-kα radiation with a wavelength of 0.1789 nm. The size of ZnO NPs was estimated from the X-ray diffraction pattern of ZnO/h-BN heterostructures by measuring the full width at half maximum (FWHM) of non-overlapping (103) ZnO peak. The Debye–Scherrer equation for calculating particle size is:

(1) $$D=\frac{K\lambda }{\beta \cos\theta },$$

where *K* is a shape factor, *λ* is the X-ray wavelength, β is the peak broadening at FWHM, and *θ* is the Bragg angle. The chemical composition and structure of the heterostructures and raw materials were analyzed by Fourier transform infrared (FTIR) and Raman spectroscopy on Bruker Vertex 70 spectrometer (Bruker, Billerica, MA, USA) and Thermo DXR spectrometer (Thermo Fisher Scientific, Waltham, MA, USA), respectively. Photoluminescence was studied using a Cary Eclipse fluorescence spectrophotometer (Agilent Technologies, Santa Clara, CA, USA) at a wavelength of 325 nm. For PL study, the concentrations of ZnO/*h*-BN in IPA was 0.3 mg/mL. ZnO NPs sols was studied as synthesized.

### 2.4. Quantum-Chemical Calculations

Quantum-chemical calculations were performed within the density functional theory (DFT) [13,14] using the VASP program package [15,16,17]. The exchange–correlation functional was calculated using the generalized gradient approximation (GGA) in the Perdew–Burke–Ernzerhof (PBE) parameterization [18]. The projector-augmented wave (PAW) [19] basis set technique was used with an energy cutoff of plane waves equal to 520 eV. We use the DFT+U formalism in the Dudarev approach [20] with parameters Ud = 10 eV and Up = 7 eV [21] to consider strong correlations between neighboring PAW spheres. The first Brillouin zone was sampled according to the Monkhorst–Pack scheme [22], and a 2 × 2 × 1 k-point mesh was chosen. To consider the van der Waals interaction, the Grimme correction (DFT-D3) [23] was applied. A vacuum region of at least 15 Å was chosen to avoid artificial interaction between periodic images of structures. To solve the problem associated with two non-equivalent (0001) wurtzite ZnO (*w*ZnO) surfaces, we used the conventional scheme, according to which the O-terminated side of *w*ZnO was covered with 1/2-valence pseudo hydrogen atoms. This scheme has been successfully applied to many semiconductors and dielectrics including *w*ZnO [24,25]. The atomic structure was visualized by the VESTA 3 [26] software. 

To create an interface between the *h*-BN and *w*ZnO structures, the “Heterotool” program developed by Sukhanova was used. The selected heterostructure unit cell consisted of ${\vec{a}}_{hBN}$×$\sqrt{7}{\vec{a}}_{hBN}$ *h*-BN and $2\sqrt{7}{\vec{a}}_{ZnO}$×$2{\vec{a}}_{ZnO}$ ZnO supercells, in which the mismatch of the individual component vectors calculated relative to the *h*-BN layer was less than 1.7%. The transformation of ZnO and *h*-BN slabs in the resulting heterostructure can be carried out using the matrix P:

(2) $$\left(a^{\prime },b^{\prime }\right)=\left(a,b\right)P=\left(a,b\right)\left(\begin{array}{cc} P_{11} & P_{12}\\ P_{21} & P_{21} \end{array}\right),$$

where $\left(a^{\prime },b^{\prime }\right)$ are the vectors of the heterostructure components (either *h*-BN or *w*ZnO), (a,b) are the initial vectors of *h*-BN or *w*ZnO. The transformation matrix P is equal to $\left(\begin{array}{cc} 0 & 1\\ 7 & -2 \end{array}\right)$ for *h*-BN and $\left(\begin{array}{cc} -6 & 2\\ -4 & 2 \end{array}\right)$ for *w*ZnO slabs in the considered heterostructure.

## 3. Results

### 3.1. TEM

**Figure 2:**
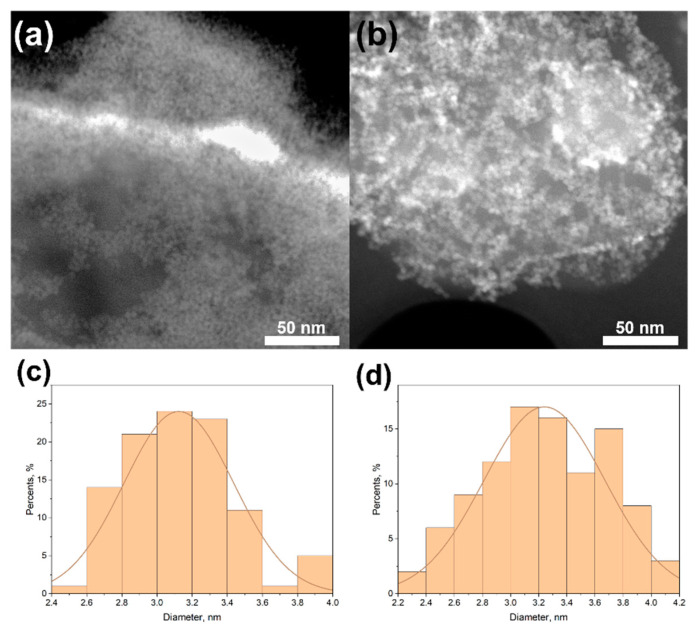
Characteristic STEM micrographs and ZnO NPs size distributions of ZnO/*h*-BNμm (**a**,**c**) and ZnO/*h*-BNnm (**b**,**d**) heterostructures.

Characteristic STEM micrographs of ZnO/*h*-BN heterostructures and size distributions of ZnO NPs are presented in Figure 2. As can be seen, size distributions of ZnO NPs deposited on *h*-BNµm and *h*-BNnm are quite the same. ZnO NPs are uniformly deposited on both carriers. According to the EDXS analysis (not shown), the mass fraction of ZnO NPs was about 25% for both ZnO/*h*-BNµm and ZnO/*h*-BNnm composites.

**Figure 3:**
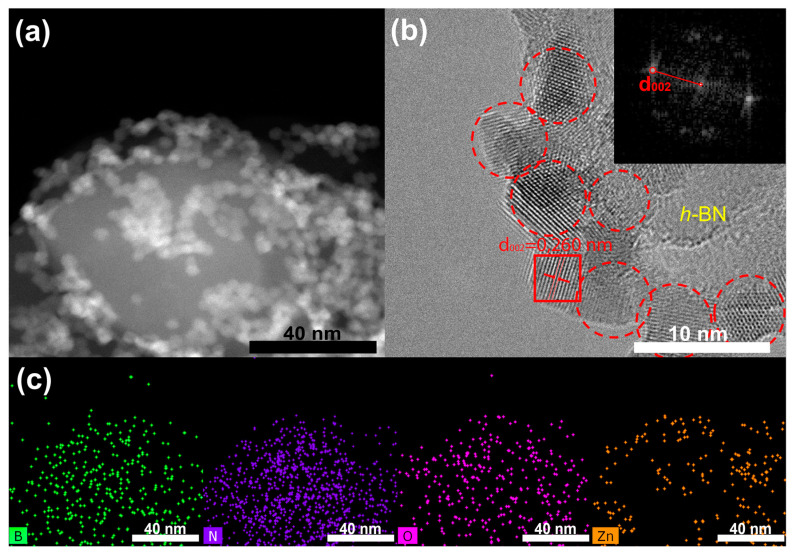
HADF-STEM image (**a**) with corresponding spatially resolved B, N, O and Zn EDXS elemental maps (**c**) and HRTEM image (**b**) of ZnO/h-BNnm sample. Insets in (**b**) show FFT pattern obtained from the framed area.

Figure 3 shows the high-angular dark field scanning TEM (HADF-STEM) image with corresponding EDXS elemental maps and HRTEM image of ZnO/*h*-BNnm sample. According to the STEM and EDXS analysis, the *h*-BN surface is covered with numerous Zn-containing NPs. The characteristic distance between the lattice fringes of these NPs is d = 0.260 nm (Figure 3b), which corresponds well to the (002) interplanar spacing in hexagonal ZnO (d = 0.2603 nm, JCPDS card No. 361451).

### 3.2. XRD and FTIR

**Figure 4:**
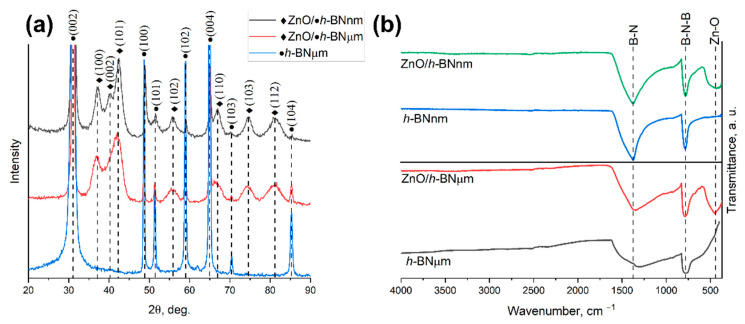
(**a**) XRD patterns and (**b**) FTIR spectra of *h*-BNμm, *h*-BNnm, ZnO/*h*-BNμm and ZnO/*h*-BNnm samples.

The results of XRD analysis are shown in Figure 4a. Compared to the reference sample *h*-BNµm, the XRD patterns of ZnO/*h*-BN composites reveal characteristic peaks of hexagonal ZnO. The size of ZnO NPs, estimated by the Debye–Scherrer formula, is 3–5 nm, which is in good agreement with the results of SEM and TEM analysis. The FTIR spectra of samples *h*-BNμm and *h*-BNnm (Figure 4b) show two characteristic *h*-BN peaks at positions 804 and 1386 cm^−1^. The presence of these peaks is associated with out-of-plane B-N-B bending and in-plane B-N stretching vibrations, respectively. The FTIR spectra of ZnO/*h*-BNμm and ZnO/*h*-BNnm composites show an additional peak approximately at 450 cm^−1^, which is a characteristic of Zn-O stretching vibration. Since no (-COOH) related peaks in the range of 1100–1700 cm^−1^ are seen, it is reasonable to conclude that the obtained ZnO/*h*-BN heterostructures do not contain residual acetate precursor or additional functional groups.

### 3.3. Photoluminescence

**Figure 5:**
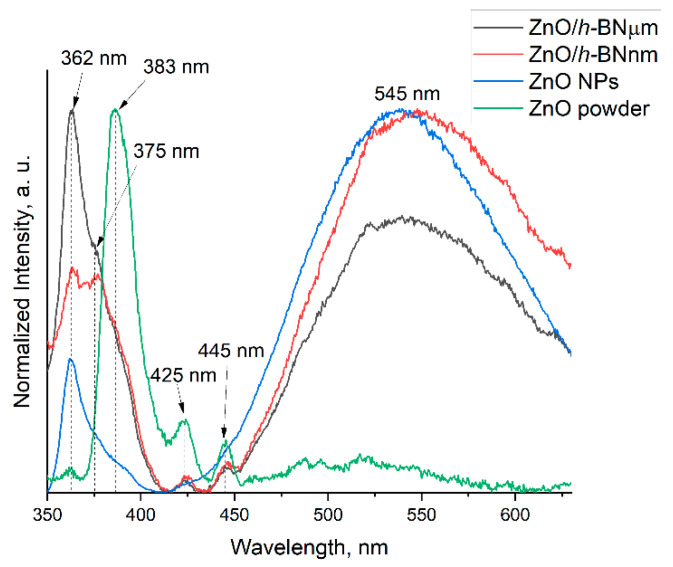
PL spectra of ZnO/*h*-BN samples in comparison with ZnO micron powder and ZnO NPs.

To study the optical properties of ZnO NPs deposited on *h*-BN support, room-temperature PL spectra of ZnO/*h*-BN heterostructures, ZnO NPs and ZnO powder were recorded and normalized (Figure 5). 

Samples show emission in three ranges: UV band with peaks at ~362 nm (~3.4 eV), ~375 nm (~3.3 eV), and ~383 nm (~3.2 eV), blue emission band with peaks at ~425 nm (~2.9 eV) and ~445 nm (~2.8 eV), and green emission band with a broad peak around ~545 nm (~2.3 eV). The emission in the UV range is attributed to the NBE due to the recombination of the free exciton transition. The blue band is due to the transition of an electron from the shallow donor level of interstitial zinc atoms (Zn_i_) to the top of the valence band (VB). The green emission is usually associated with oxygen vacancies (V_o_). A more detailed description of the origin of each peak, with references to literature data, is given in Table 1.

As expected, due to the large particle size, the micron ZnO powder shows a stronger peak in the UV region, while the as-synthesized ZnO NPs mainly emit in the visible region. Samples ZnO/*h*-BNµm and ZnO/*h*-BNnm exhibit relatively strong emissions in both the UV and visible regions. The most interesting observation is that, compared to ZnO NPs, the PL of samples ZnO/*h*-BNµm and ZnO/*h*-BNnm in the UV range is much stronger. It is noteworthy that although the size of ZnO NPs in samples ZnO/*h*-BNµm and ZnO/*h*-BNnm is approximately the same, ZnO/*h*-BNµm emits much more strongly in the UV range, but is weaker in the visible range.

## 4. Discussion

**Figure 6:**
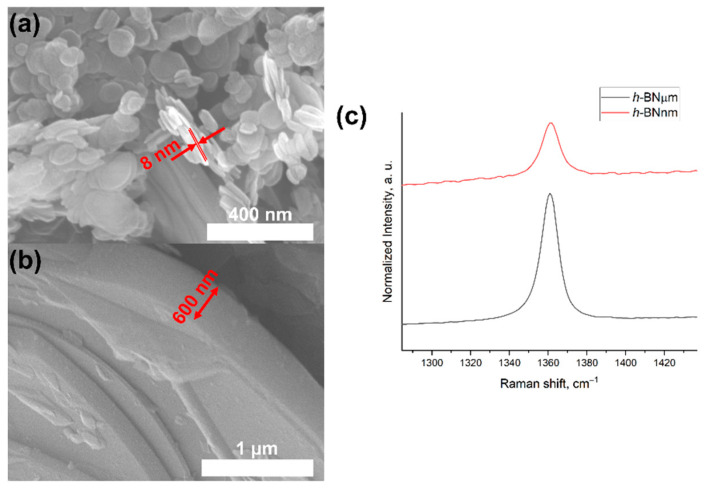
SEM images of *h*-BNnm (**a**), *h*-BNµm (**b**) and their Raman spectra (**c**).

Considering that the sizes of ZnO NPs in the ZnO/*h*-BNµm and ZnO/*h*-BNnm samples are approximately the same, there must be another reason for their different PL intensity in the UV range, rather than surface-to-volume ratio. The most probable explanation for the weakening of DLE and the enhancement of NBE can be the particle/support interaction, which leads to a change in the electronic structure of the metal oxide NPs [3,4]. Visible light emission arises due to the recombination of an electron from the conduction band with a double positively charged oxygen vacancy. To generate Vo^++^, it is necessary to activate a single positively charged oxygen vacancy (Vo^+^). This requires that a hole trapped in surface defects tunnels to Vo^+^, forming Vo^++^. This process is faster than the exciton recombination responsible for the NBE, but if the local electronic structure of the ZnO surface is changed with a dielectric, there will be a decrease in the number of surface traps due to the screening effect, which will weaken the DLE and enhance the NBE. Since the surface of ZnO NPs is passivated because the contacting material has a different dielectric constant, it may be the difference in dielectric constant between *h*-BNnm and *h*-BNµm that caused the effect we observed. The dielectric constant, in turn, can depend on the thickness of *h*-BN particles [33], which in our case is an order of magnitude larger for *h*-BNµm compared to *h*-BNnm, as evidenced by the results of SEM analysis and an increase in the intensity of the Raman peak [34] (Figure 6). 

**Figure 7:**
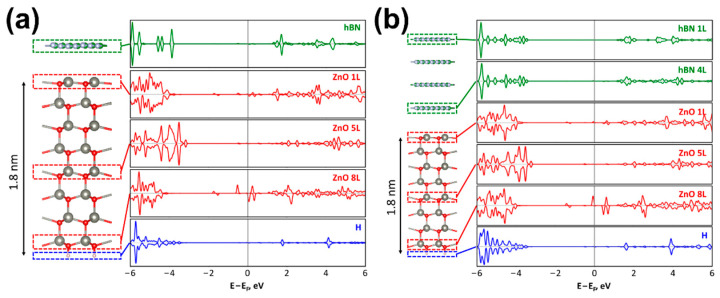
1L-*h*-BN/*w*ZnO (**a**) and 4L-*h*-BN/*w*ZnO (**b**) heterostructures and corresponding densities of states. Red, large grey, small grey, green, and pink cycles show oxygen, zinc, boron, nitrogen, and hydrogen atoms.

The quantum chemical calculations were performed to explain the effect of the *h*-BN thickness on the electron density redistribution in the *w*ZnO/*h*-BN heterostructure. Two systems were considered with a ~1.8 nm thick *w*ZnO slab (1L-*h*-BN/*w*ZnO) and either one or four *h*-BN layers in the AA’ stacking (4L-*h*-BN/*w*ZnO). The choice of this stacking is due to the fact that previous studies have shown that this stacking is the one of the most energetically favorable [35,36]. The calculated densities of states (DOSs) of the 1L-*h*-BN/*w*ZnO and 4L-*h*-BN/*w*ZnO heterostructures demonstrate the difference near the Fermi level of *w*ZnO, and in both cases the DOSs depend on the distance from the considered *w*ZnO layer to the interface with *h*-BN (Figure 7a,b). 

**Figure 8:**
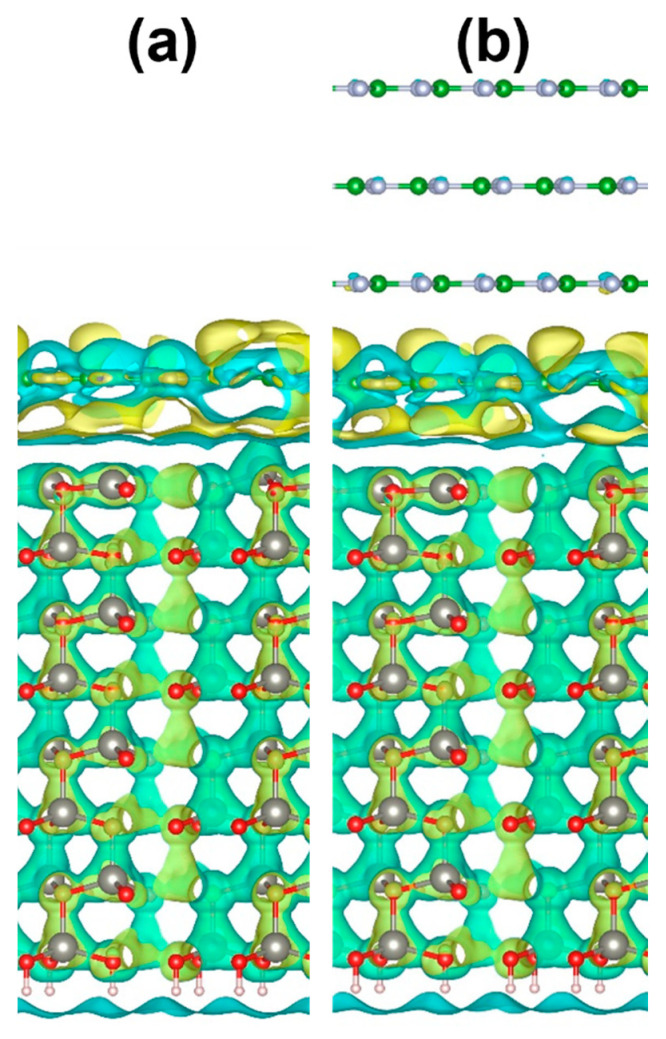
Charge difference distribution between h-BN and wZnO for (**a**) 1L-hBN/wZnO and (**b**) 4L-hBN/wZnO heterostructures. Yellow and blue clouds show the accumulation and loss of electrons. The isosurface level is 5 × 10^−5^ e/Å^3^.

The charge difference distribution calculated as:

(3) ρ=ρ(wZnO/h-BN)−ρ(h-BN)−ρ(wZnO)

shows the appearance of hybridization between the orbitals of the *h*-BN monolayer closest to the interface and the upper *w*ZnO layer (Figure 8).

To study the optical properties of *w*ZnO/*h*-BN composites, we considered the wavelength-dependent complex dielectric function

(4) $$\varepsilon \left(\lambda \right)=\varepsilon_{1}\left(\lambda \right)+i\varepsilon_{2}\left(\lambda \right),$$

in which the real part was calculated using the Kramers–Kronig transformation, and the imaginary part was determined by the sum over empty states [37,38]. The extinction coefficient in the direction parallel and perpendicular to the heterostructure surface was calculated as:

(5) $$K\left(\lambda \right)={\left[\frac{\sqrt{\varepsilon_{1}^{2}\left(\lambda \right)+\varepsilon_{2}^{2}\left(\lambda \right)}-\varepsilon_{1}\left(\lambda \right)}{2}\right]}^{1/2}.$$

**Figure 9:**
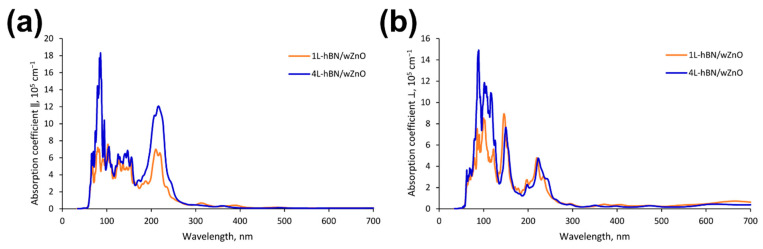
Dependence of the extinction coefficient of h-BN/*w*ZnO heterostructures on the irradiation wavelength in (**a**) transverse and (**b**) perpendicular directions to the surface.

**Figure 10:**
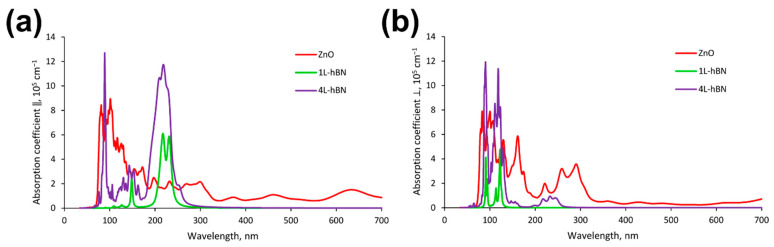
Dependence of the extinction coefficient of individual components of wZnO/h-BN heterostructures on the irradiation wavelength in (**a**) transverse and (**b**) perpendicular directions to the surface.

The obtained results for the *w*ZnO/*h*-BN heterostructures are shown in Figure 9, and the individual parts of the heterostructures are depicted in Figure 10. When *w*ZnO/*h*-BN composites are irradiated with light with parallel and perpendicular polarization, three groups of absorption peaks are observed in the UV region, located approximately at 85, 140, and 215 nm (transverse polarization) and 100, 150, and 239 nm (perpendicular polarization). It should be noted that the intensity of the absorption peaks for the 4L-*h*-BN/*w*ZnO heterostructure is higher than that for 1L-*h*-BN/*w*ZnO, which agrees well with experimental results.

**Figure 11:**
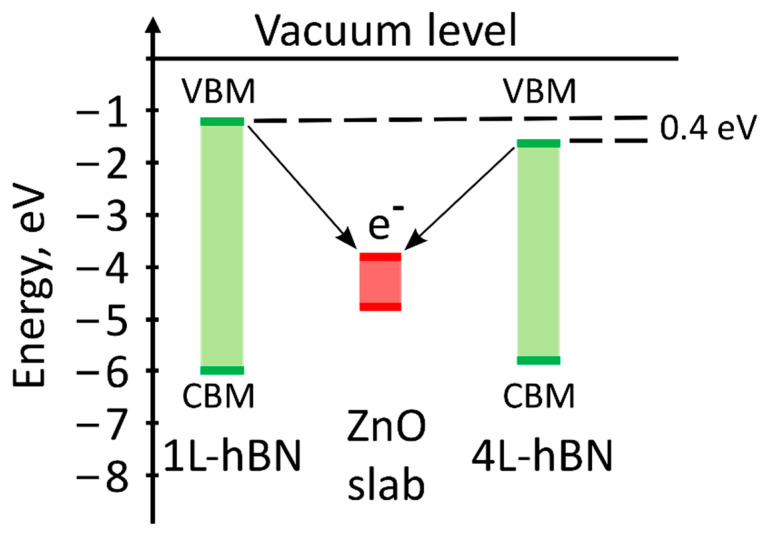
Schematics of band gap arrangement of freestanding 1L-*h*-BN and 4L-*h*-BN relative to *w*ZnO slab.

The *h*-BN band gap in the monolayer exceeds the value for the bulk form [39]. According to previous atomistic calculations [40], multilayered *h*-BN, despite the indirect band gap, luminesces as strongly as direct band gap materials. Therefore, epitaxial *h*-BN has a high potential in deep ultraviolet optoelectronics and quantum photonics. The band gap arrangement of freestanding 1L-*h*-BN and 4L-*h*-BN relative to the *w*ZnO slab is shown in Figure 11.

Despite the significant difference in the calculated band gap values for the *w*ZnO slab and *h*-BN, the position of the valence band minimum (VBM) of the upper layer of the *w*ZnO slab is lower by 2.0 and 2.4 eV than the VBM of 4L- and 1L-*h*-BN, respectively. The VBM of the 4L-*h*-BN is 0.4 eV closer to the VBM of *w*ZnO, which is consistent with the observed changes in the PL spectra and enhancement in the UV region for thicker *h*-BN.

## 5. Conclusions

We have successfully deposited 3–5 nm ZnO nanoparticles (NPs) on two types of *h*-BN support with sizes of about 100 nm and 1 µm. The *h*-BN support enhances the photoluminescence of ZnO NPs in the UV range. When using micron-sized *h*-BN carriers, the PL in the UV range is stronger, and it is weaker in the visible range compared to nano *h*-BN support. This may be because *h*-BN, as an insulator, passivates the surface of ZnO NPs. We assume that the degree of passivation may depend on the thickness of the *h*-BN particles, which affects the electronic structure of the ZnO NPs surface. This is confirmed by the results of DFT calculations, which indicate the appearance of hybridization at the *h*-BN/*w*ZnO interface and an increase in the intensity of absorption peaks with increasing *h*-BN thickness. Thus, we have proposed a simple and scalable method for creating ZnO/*h*-BN heterostructures, which makes it possible to control the PL properties of ZnO NPs by changing the thickness of *h*-BN support.

## Figures and Tables

**Table 1:** Possible origin of emission peaks in PL spectra of ZnO/*h*-BN samples.

nm(eV)	Electron Transition	References
~363(~3.4)	CB ^1^→VB	[27,28]
~375(~3.3)		[29]
~383(~3.2)		[30,31]
~425(~2.9)	Zn_i_→VB	[5]
~445(~2.8)	V_Zn_^++ 2^→VB	[32]
~545(~2.3)	CB→V_O_^++ 3^	[29]

^1^ CB—conduction band. ^2^ V_Zn_^++^—ionized zinc interstitials. ^3^ V_O_^++^—double positively charged oxygen vacancy.

## Data Availability

Data are available from the corresponding author upon reasonable request.
